# A Cell-Based Model of Extracellular-Matrix-Guided Endothelial Cell Migration During Angiogenesis

**DOI:** 10.1007/s11538-013-9826-5

**Published:** 2013-03-15

**Authors:** Josephine T. Daub, Roeland M. H. Merks

**Affiliations:** 1 Centrum Wiskunde & Informatica, Science Park 123, 1098 XG Amsterdam, The Netherlands; 2 Section Computational Science, Informatics Institute, University of Amsterdam, Science Park 904, 1098 XH Amsterdam, The Netherlands; 3 Present Address: Institute of Ecology and Evolution, University of Bern, Baltzerstrasse 6, 3012 Bern, Switzerland; 4 Swiss Institute of Bioinformatics, 1015 Lausanne, Switzerland; 5 Department of Ecology and Evolution, University of Lausanne, 1015 Lausanne, Switzerland; 6 Netherlands Consortium for Systems Biology and Netherlands Institute for Systems Biology, Science Park 123, 1098 XG Amsterdam, The Netherlands; 7 Mathematical Institute, University Leiden, P.O. Box 9512, 2300 RA Leiden, The Netherlands

**Keywords:** Angiogenesis, Extracellular matrix, Cellular Potts model, Branching growth, MMPs

## Abstract

Angiogenesis, the formation of new blood vessels sprouting from existing ones, occurs in several situations like wound healing, tissue remodeling, and near growing tumors. Under hypoxic conditions, tumor cells secrete growth factors, including VEGF. VEGF activates endothelial cells (ECs) in nearby vessels, leading to the migration of ECs out of the vessel and the formation of growing sprouts. A key process in angiogenesis is cellular self-organization, and previous modeling studies have identified mechanisms for producing networks and sprouts. Most theoretical studies of cellular self-organization during angiogenesis have ignored the interactions of ECs with the extra-cellular matrix (ECM), the jelly or hard materials that cells live in. Apart from providing structural support to cells, the ECM may play a key role in the coordination of cellular motility during angiogenesis. For example, by modifying the ECM, ECs can affect the motility of other ECs, long after they have left. Here, we present an explorative study of the cellular self-organization resulting from such ECM-coordinated cell migration. We show that a set of biologically-motivated, cell behavioral rules, including chemotaxis, haptotaxis, haptokinesis, and ECM-guided proliferation suffice for forming sprouts and branching vascular trees.

**Electronic Supplementary Material:**

The online version of this article (doi:10.1007/s11538-013-9826-5) contains supplementary material, which is available to authorized users.

## Introduction

The outgrowth of new blood vessels from preexisting vessels, called angiogenesis, is a crucial step in many physiological and pathological mechanisms, including wound healing and tumor growth. Once cells in a tissue, e.g., a growing tumor, are short in oxygen or nutrients, they secrete a range of angiogenic growth factors (De Smet et al. 2009), including vascular-endothelial growth factor (VEGF). VEGF activates endothelial cells (ECs), the cells forming the inner lining of blood vessels, resulting in increased cell survival, migration, and proliferation. The activated ECs differentiate into stalk and phalanx cells—forming the body of the sprout—and a tip cell phenotype that migrates chemotactically toward the source of VEGF (De Smet et al. 2009). Initially, VEGF stimulates filopodial extensions of specialized ECs, called tip cells (Gerhardt et al. 2003). The sprout grows out as the ECs further down in the sprout proliferate (Gerhardt et al. 2003). Angiogenesis is a topic of intensive experimental investigation so its phenomenology and the molecular signals contributing to it have been well characterized (Carmeliet 2005; Carmeliet and Jain 2000; Folkman 2007). Yet it is poorly understood how the biological components fit together dynamically to drive the outgrowth of blood vessels.

A key process in angiogenesis is cellular self-organization. ECs cultured in vitro autonomously organize into vascular networks (Folkman and Hauenschild 1980); and ECs move along growing sprouts (Jakobsson et al. 2010). Thus, an important question becomes what (genetically regulated) cell behaviors and cellular responses are responsible for the self-organization of endothelial cells into blood vessel sprouts. To answer this question, cell-based computational models take as *input* a set of measurable and quantifiable behaviors and the responses of individual cells to chemical and mechanical cues from the microenvironment. The *output* of the model is a prediction of the resulting collective behavior of the cells that was not explicitly prescribed, e.g., the formation of a sprout, sprout branching, and sprout anastomosis (Merks and Glazier 2005; Merks and Koolwijk 2009; Anderson et al. 2007). In this way, previous cell-based models have predicted potential mechanisms for the formation of vascular networks (Merks et al., 2004, 2006; Szabo et al., 2007, 2008; Guidolin et al., 2009; Scianna et al., 2011; Köhn-Luque et al., 2011) and angiogenic sprouting (Bauer et al. 2007, 2009; Merks et al. 2008; Szabo et al. 2008). Thus, a cell-based model allows us to mechanistically dissect the workings of a biological mechanism in a *predictive* fashion, because each of the model assumptions corresponds with a biological component accessible to experimental manipulation.

This cell-based approach to computational modeling of angiogenesis contrasts with a number of previous, descriptive models of angiogenesis (e.g., Anderson and Chaplain, 1998; Milde et al., 2008; Sun et al., 2005; Owen et al., 2009; Perfahl et al., 2011; Watson et al., 2012). Although such models successfully simulate many phenomena associated with angiogenesis, a problem is that model *input* and model *output* are not always strictly separated: In addition to rules for tip cell motility, these models include explicit, descriptive rules for tip branching, anastomosis, and specific assumptions to prescribe the length of the branches and sometimes branching angles. Descriptive models are very helpful because they can integrate our current experimental knowledge of a developmental mechanism, but—in contrast to a cell-based model—they cannot help dissect and integrate the underlying molecular and cellular mechanisms responsible for the mechanisms they prescribe explicitly. The agent-based models of Bentley and coworkers do take a predictive approach, like cell-based models, focusing on the molecular and cellular signaling mechanisms responsible for tip and stalk cell selection (Bentley et al., 2008, 2009). This model has suggested a novel, filopodia-mediated tip-cell selection mechanism, the molecular level of the model is experimentally plausible, and it has guided experimental studies (Jakobsson et al. 2010; Guarani et al. 2011). However, because in this model cells cannot move relative to one another, the model is not suited for our purpose: studying cellular self-organization in which cell motility is a key process.

Although cell-based simulation studies show how collective cell behavior can produce vascular networks and angiogenic sprouts, many lack a detailed description of the extracellular matrix (ECM), the jelly or hard materials that cells secrete. The ECM provides mechanical support to endothelial cells, and mediates signaling via secreted molecules (Hynes 2009) and mechanical strains (Reinhart-King et al. 2008) between cells (Davis and Senger 2005). Cells can pick up molecular signals in the ECM long after another cell left it there. Mechanical signals, in the form of tissue strains and stresses to which cells respond (Mammoto et al. 2009), can act over long distances and integrate mechanical information over the whole tissue (Nelson et al. 2005). Thus, apart from providing structural support, the ECM is key to cellular coordination, because (a) it stores cellular signals over a long time, or it is such a cellular signal itself, and (b) it integrates biomechanical information over long distances. Thus, the ECM is a key component of the microenvironment that endothelial cells live in and cannot be ignored in computational models.

The cell-based models by Bauer and coworkers (Bauer et al., 2007, 2009) focus on the role of the ECM as an obstacle and directional guidance cue for EC migration. Representing ECM fibers as static obstacles they demonstrate how ECM fibers distributed in uniform or directionally-biased random orientations can direct the migration of a growing angiogenic sprout. Also, their model shows how local variations in ECM density can induce sprout splitting (Bauer et al. 2007). In their model of in vitro vasculogenesis, Köhn-Luque and coworkers (Köhn-Luque et al. 2011) focus on the role of the ECM as a storage of growth factors, showing how endothelial cells can induce local gradients of chemoattractants by secreting proteolytic enzymes that locally release ECM-bound VEGF molecules. Apart from these models based on the Cellular Potts method (CPM), the partial-differential equation (PDE) models of vasculogenesis by Manoussaki and coworkers (Manoussaki et al., 1996, 2003) and those of Tracqui and coworkers (Namy et al. 2004) study the ECM as a medium for biomechanical signaling. Thus, previous cell-based models have studied the function of the ECM as a barrier for cell migration (Bauer et al., 2007, 2009) or as an local inactivating storage for chemotactic signals that proteolytic enzymes release (Köhn-Luque et al. 2011).

Because cells can digest the ECM and secrete new ECM materials, the ECM can also function as a “written” cellular signal, that “records” previous positions of the ECs and facilitates the motility of subsequent ECs. Several variants of such *facilitated random walk* mechanisms were proposed for angiogenesis. Yin and coworkers (Yin et al. 2008) showed that cells deposit collagen in microfluidic devices. This produces tracks that other ECs can follow by altering their velocity in response to the collagen trail collagen. They also found that stimulation by VEGF inhibits the track-following behavior of the ECs. An agent-based model demonstrated that this VEGF-inhibited track-following behavior produces vascular trees with a typical “brush-border”-effect (enhanced branching) near a source of VEGF, e.g., a tumor. Levine (2001a, 2001b) and Plank and Sleeman (2003) proposed that tip cells locally secrete proteolytic enzymes that digest the ECM and allow the tip cell to pass through. A similar approach was taken by Anderson and Chaplain (1998). They proposed a continuous model for tumor-induced angiogenesis, and derived from that a discrete, stochastic model simulating the motility of ECs. Their model simulates migration of endothelial cells from a parent vessel toward a tumor, which chemotact along a gradient of angiogenic growth factors that the tumor produces. The ECs interact with the surrounding extracellular matrix by breaking down and secreting fibronectin, an extracellular matrix component. The ECs’ migration is biased toward higher fibronectin concentrations, a process called haptotaxis; as a result, the ECs spread into “fresh” fibronectin by breaking it down locally and migrating to a nearby location with higher fibronectin concentration. We have recently introduced a computational model of sprout formation in an in vitro assay of angiogenic sprouting from endothelial monolayers in fibrin matrices (Boas et al. 2013). In that model, fibrin acts as an obstacle for cell migration. Tip cells secrete uPA that degrades fibrin so cells can migrate into the fibrin. In the present paper, we further explore the effect of proteolysis-based cell-ECM interactions in a cell-based model, describing the behavior of both the endothelial cells at the tip and the trailing endothelial cells. In contrast to the model by Boas et al., we here represent the ECM with a continuous field, and model the effect of the ECM on cell motility in more detail.

The ECM can affect cell motility in at least two ways. First, ECs can follow local gradients, crawling to higher concentrations of the ECM, a process called haptotaxis (Lamalice et al. 2007). Second, ECs can increase or reduce their motility in response to the absolute concentrations of ECM, a mechanism called *haptokinesis*. Typically, cell speed, spreading, and membrane activity is maximal at intermediate levels of ECM densities, both on 2D substrates (Chon et al. 1998; Cox et al. 2001; DiMilla et al. 1993; Gaudet et al. 2003; Palecek et al. 1997; Wu et al. 1994) and in 3D matrices (Zaman et al. 2006).

To generate ECM gradients, ECs locally degrade or deposit matrix proteins (e.g., fibronectin and collagen). After VEGF-stimulation, ECs produce diffusing and membrane-bound proteolytic enzymes, among which are the matrix metalloproteinases (MMPs) that can proteolytically degrade almost every ECM protein. The membrane bound MT-MMP is a key player in this process, breaking down ECM components close to the tip of the sprout, and inducing the release of other MMPs like MMP2 (Pepper 2001; van Hinsbergh and Koolwijk 2008).

Could such signaling via a nondiffusing ECM coordinate collective cell behavior during angiogenesis? To address this question, we constructed a hybrid CPM-PDE model, based on the following, biologically plausible assumptions: (1) Tumors secrete VEGF resulting in a VEGF gradient (Folkman 2007); (2) VEGF induces secretion of diffusive MMPs by endothelial cells. (3) MMPs degrade ECM components near the cell surface. (4) ECs move along VEGF gradients (Gerhardt 2008; Gerhardt et al. 2003) and they (5) migrate toward higher ECM densities (Lamalice et al. 2007; Senger et al. 2002). (6) Cell speed and spreading are optimal at intermediate ECM densities and (7) cells proliferate if a large part of their surface is in contact with the ECM (Ausprunk and Folkman 1977; Coomber and Gotlieb 1990). In the present model, we ignore the differentiation of ECs into tip and stalk cells (Gerhardt et al. 2003; De Smet et al. 2009; Phng and Gerhardt 2009). With these assumptions, our model is probably most similar to the model of Anderson and Chaplain (1998), who considered assumptions (1)–(5). New in our model are assumptions (6) and (7). Also, our model does not need any additional rules for branching or anastomosis. All the angiogenesis-like phenomena reported here are exclusively due to assumptions (1)–(7). We show that these suffice for robustly branched vascular trees, in the absence of explicit model rules for vascular branching. In the remainder of this paper, we first describe our model and illustrate the contribution of each of the assumptions to producing a vascular tree. Next, we provide a thorough parameter analysis. We end by discussing the biological relevance of our model observations and discussing future directions.

## Model Setup

**Fig. 1:**
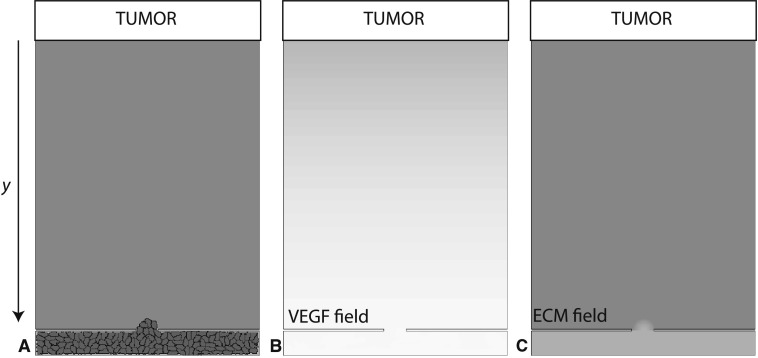
Setup of model domain. **A** Initial cellular Potts model configuration (endothelial cells) and extracellular matrix field. To mimic an early stage vessel sprout, 125 cellular Potts cells, each consisting of approximately 50 lattice sites are positioned behind a digested vessel wall, on top of a uniform ECM concentration. **B** Steady state VEGF field; **C** Initial ECM field

We model endothelial cells using a Cellular Potts model (CPM) (Glazier and Graner 1993) (aka Glazier–Graner–Hogeweg model), a lattice-based cell-based modeling technique that represents cells as a connected domain of square lattice sites. It simulates stochastic cell motility by iteratively expanding and contracting the domains, depending on a set of cell behavior rules; see Sect. 2.1 for a detailed description. We use a partial-differential equation (PDE) description for fields of extracellular matrix materials, proteolytic enzymes, and diffusing growth factors. The model domain is similar to that used by previous authors (Anderson and Chaplain 1998; Yin et al. 2008): in a rectangular dish of 250×300 lattice units (corresponding to approximately 500 μm×700 μm) 125 endothelial cells are placed behind a vessel wall situated at the bottom of the dish. The cells can migrate toward the top of the dish through a gap of 25 lattice units in the wall in the direction of a tumor, which we assume to be located beyond the top of the dish (Fig. 1A). We define only one type of cell, so we do not distinguish tip cells and stalk cells. A set of three PDEs describes the concentrations of VEGF, MMPs and ECM components. We solve the PDE for VEGF analytically, and solve the two coupled PDEs for MMPs and ECM concentration numerically.

### Cellular Potts Model

**Fig. 2:**
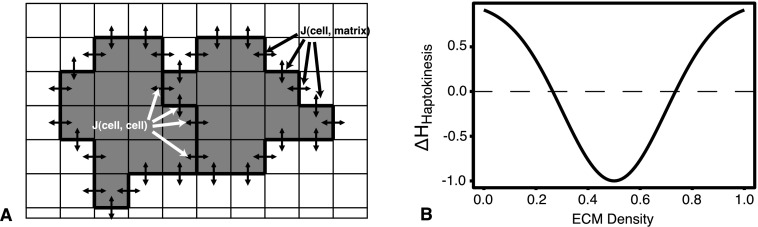
Cellular Potts model and interaction with extracellular matrix. **A** Lattice with two adjacent endothelial cells (*grey*), surrounded by an extracellular matrix (*white*). *Double arrows* represent cell–cell and cell-ECM adhesive bonds. For simplicity, diagonal bonds are not shown. **B** Haptokinesis energy term (Eq. (8)) for *η*=1, *μ*=0.5, and *ρ*=0.2

The CPM represents biological cells as patches of lattice sites. Each cell has a unique index $\sigma\in\mathbb{N}$, which is assigned to every lattice site that is occupied by that cell. The type of a cell *σ* is denoted with $\tau(\sigma)\in\mathbb{N}$. The extra-cellular matrix (ECM) consists of all lattice sites not occupied by cells and is labeled with index *σ*=0 and type *τ*=0. The interfaces between adjacent lattice sites $\vec{x}$ and $\vec{x}'$ with unequal index $\sigma_{\vec{x}}\neq\sigma_{\vec{x}'}$ represent membrane bonds, with an associated, cell-type dependent adhesion energy given by $J(\tau(\sigma_{\vec{x}}), \tau (\sigma_{\vec{x}'}))$ (Fig. 2A). An area constraint penalizes cell shapes deviating too much from their preferred area; to drive cell elongation we use a length constraint, which penalizes cell shapes shorter or longer than a target length (Merks et al. 2006). The ECM has no area or length constraint.

The “effective energy” is given with the CPM Hamiltonian: 
1 where $(\vec{x},\vec{x}')$ is a pair of adjacent lattice sites, *δ* is the Kronecker delta, *A*(*σ*) is the current area of cell *σ*, *A*_*T*_(*σ*) is its target area, and *λ*_*A*_ is an area elasticity parameter; similarly, *L*(*σ*) represents the current length of cell , *L*_*T*_(*σ*) is its target length and *λ*_*L*_ is the strength of the length constraint. The optimal way to minimize both the length constraint and adhesion energies together is to split the cell into two rounded patches; we therefore require an additional connectivity constraint. The cell length estimation method and connectivity constraint algorithm are described in detail elsewhere (Merks et al. 2006).

To mimic membrane extensions and retractions, we repeatedly attempt to replace the index $\sigma_{\vec{x}}$ of a random lattice site $\vec{x}$ by the index $\sigma_{\vec{x}}'$ of one of its random adjacent sites $\vec{x}'$. We calculate Δ*H*, the change in total effective energy *H* that would occur if we performed the copy, and accept the attempt with Boltzmann probability:

2 $$ P(\Delta H) = \begin{cases} e^{-{\Delta H}/{M}}& \text{if $\Delta H \geq0$},\\ 1 & \text{if $\Delta H < 0$}, \end{cases} $$

where *M* defines the *intrinsic random motility* of the cell membranes. This allows energetically unfavorable cell movements. Note that we define cell *motility* here as the active movement of the cell’s perimeter driven by extension and retraction of pseudopods, which may or may not lead to cell *migration*.

We will set the target area *A*_*T*_(*σ*)=50 lattice sites and the target length *L*_*T*_(*σ*)=15 lattice sites. We set the adhesion energy at cell–cell borders *J*_CC_=40 and at cell–matrix borders, *J*_CM_=25, in order to make attachments between cells slightly favorable over cell–matrix bonds; hence the surface tension becomes $\gamma_{\mathrm{CM}}=J_{\mathrm{CM}}-{1\over2}J_{\mathrm{CC}} = 5 > 0$, so cells adhere to one another (Glazier and Graner 1993). The intrinsic motility parameter is set to *M*=100.

When performing a copy attempt we select the source site $\vec{x}'$ from the 20, first- to fourth-order nearest neighbors of $\vec{x}$, to improve the isotropy (Holm et al. 1991; Marée et al. 2007). During a Monte Carlo Step (MCS), we carry out *N* copy attempts where *N* is the total number of lattice sites in our dish. We define a high cell-border energy to prevent cells from adhering to the boundaries of the lattice.

**Fig. 3:**
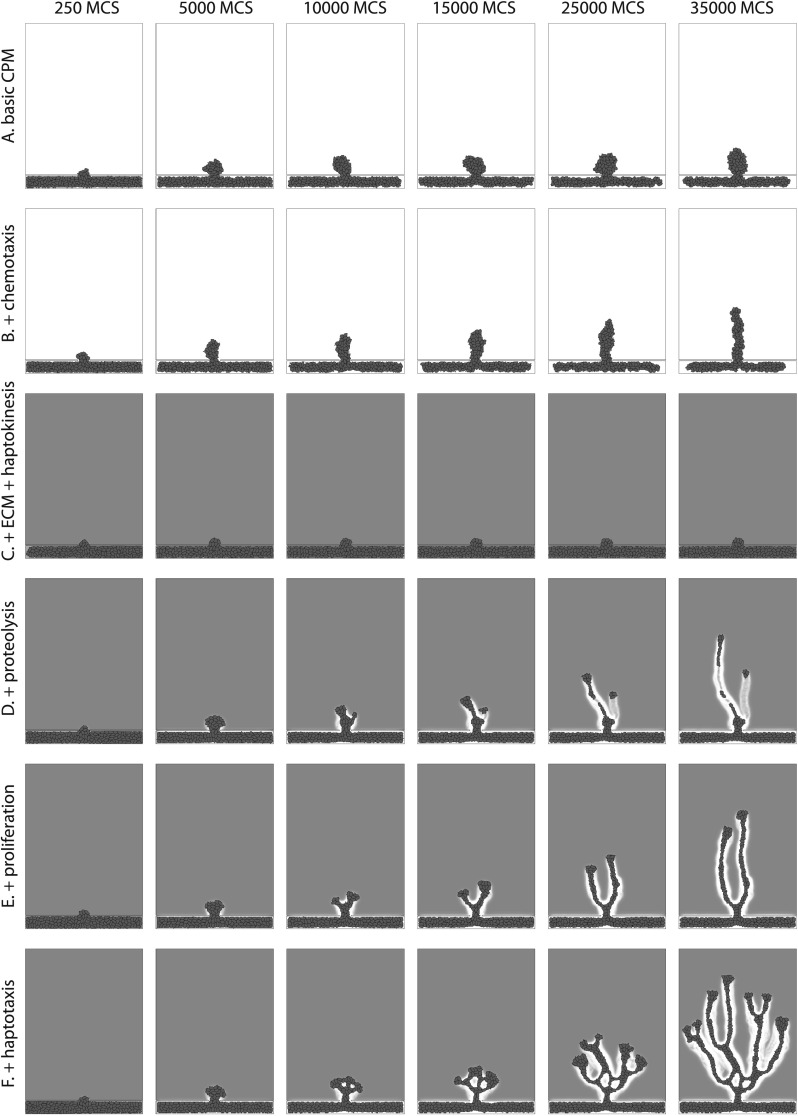
Incremental overview of effect of model components. For parameters, see Table 2. **A** Only adhesion energy and area and length constraints; **B** after adding chemotaxis along a vertical source of VEGF (Eqs. (3)–(5)); **C** after adding an initial layer of ECM and a haptokinesis rule (Eq. (8)); note that this concentration of ECM prevents cells from moving; **D** after adding proteolysis (Eqs. (6)–(7)) the cells coast along trails of intermediate ECM concentration; **E** the proliferation rule (Eq. (9)) allows cells to form connected branches; **F** haptotaxis (Eq. (12)) enhances branching. See also Supplementary Movie 1

The remainder of Sect. 2 describes the implementation of the endothelial cell behaviors that our model describes. Figure 3 summarizes the effect of each of these assumptions on the collective behavior of the simulated ECs. For example, with only the adhesion and area and length constraints described so far, cells migrate out of the parent vessel forming a clump of cells (Fig. 3A).

For simplicity and current lack of quantitative data, we use the set of unitless, reference parameter values listed in Table 1, and study their relative importance in Sect. 3. As Supplementary Text 1 demonstrates, the results hold if empirical values are used where they are known, choosing appropriate values for the remaining free parameters. However, it is unclear to what extent these free parameters can correct for imprecise values of the “known” parameter values. We therefore prefer to work with dimensionless parameter sets here, thus avoiding the impression that our model results were quantitatively valid at present. To validate our model, we instead perform parameter sensitivity analyses (Sect. 3), and compare its results with published, qualitative experiments.

**Table 1:** Unitless reference values of model parameters

Parameter	Value	Description
*χ*	5000	chemotaxis strength
*Γ*	300	haptotaxis strength
s	7.0	saturation haptotaxis
*η*	200	haptokinesis strength
*ϵ*_EM_	3×10^−3^	decay rate ECM
*α*_MV_	8×10^−5^	secretion rate MMPs
*ϵ*_*M*_	1×10^−3^	decay rate MMPs
*D*_*M*_	1×10^−14^	diffusion coefficient MMPs
*D*_*V*_	6×10^−11^	diffusion coefficient VEGF
*ϵ*_*V*_	1×10^−3^	decay rate VEGF
*ρ*_min_	0.73	threshold ratio for proliferation
*λ*_*A*_	25	parameter area constraint
*λ*_*L*_	25	parameter length constraint
$c_{E,{\rm init}}$	0.9	initial ECM density outside parent vessel
*c*_*V*_(0)	0.87	VEGF concentration near tumor
*X*	250	width of dish (lattice sites)
*Y*	350	length of dish (lattice sites)
$Y_{\rm gap}$	30	distance vessel wall from bottom of dish
$X_{\rm gap}$	25	width of gap in vessel wall (lattice sites)
$\mathit{MCS}_{\rm tot}$	40000	number of total MCS

**Table 2:** Parameter settings for the simulations shown in Fig. 3

Parameter	Value						Description
	A	B	C	D	E	F	
*χ*	0	6000	6000	6000	6000	6000	chemotaxis strength
*η*	0	0	200	200	200	200	haptokinesis strength
$c_{E,{\rm init}}$	0	0	0.9	0.9	0.9	0.9	initial ECM density
*ϵ*_EM_	0	0	0	2×10^−3^	2×10^−3^	2×10^−3^	decay rate ECM
$\rho_{\rm min}$	1	1	1	1	0.73	0.73	threshold ratio for proliferation
*Γ*	0	0	0	0	0	1000	haptotaxis strength
	Other parameters: see Table 1						

### Chemotaxis along VEGF Gradient

We define a tumor nearby the vessel, at the top of the simulation domain. We assume the tumor produces VEGF uniformly and throughout, allowing us to approximate it as a planar source at the top. The VEGF degrades at a rate proportional to the concentration, *c*_*V*_,

3 $$ {{\partial{c_V}}\over{\partial t}} = - \epsilon_V c_V + D \nabla^2 c_V $$

with *D*, the diffusion coefficient and *ϵ*_*V*_, the degradation rate of VEGF. In the simulations presented in this paper, we assume for simplicity that the gradient is at steady state, ${{\partial{c_{V}}}\over{\partial t}} =0$. We furthermore assume that the binding of VEGF to ECs is slow relative to the supply of VEGF from the tumor and the degradation, allowing us to ignore the disturbance of the VEGF-field by the ECs. We can then approximate the VEGF-field with a steady-state, analytical solution of Eq. (3), instead of solving it numerically. Assuming a constant concentration *c*_*V*_(0) at the tumor boundary and open boundary conditions at the parent vessel, the one-dimensional analytical solution of the gradient at steady state becomes

4 $$ c_V(x_2)=c_V(0)e^{-x_2/\lambda}\quad \text{with } \lambda= \sqrt{D\over \epsilon_V}. $$

If the tumor is spherical and much larger relative to the simulation domain, we can assume the tumor boundary is flat and approximate the VEGF gradient by a planar gradient according to the analytical solution in Eq. (4) (Fig. 1B).

Chemotaxis up the VEGF-gradients is incorporated by including an extra reduction in energy for extensions into the ECM toward higher concentrations of VEGF (as described in Merks et al., 2008). After we calculate the change in effective energy, Δ*H*, associated with a potential copying step according to Eq. (1), we subtract a contribution due to chemotaxis. The energy change due to chemotaxis then becomes

5 $$ {\Delta H_{\rm chemotaxis}}= -\chi\delta ( \sigma_{\vec{x}},0) \bigl(c_V(\vec{x})-c_V\bigl( \vec{x}'\bigr)\bigr) $$

where $(\vec{x},\vec{x}')$ is a pair of adjacent lattice sites, with $\vec{x}'$ the source site of the copy. $c_{V}(\vec{x})$ is the local concentration of VEGF at site $\vec{x}$, and *χ* is the strength of the chemotactic response. Note that it is not possible to include the chemotaxis term in the Hamiltonian (Eq. (1)), because it involves gradient calculations over the *direction* of copying. Since primarily the extending filopodia are able to sense and react to chemotactic cues, we consider only extensions of cells into the ECM to contribute chemotactically to the total energy (Merks et al. 2008). Including chemotactic retraction gives similar branching sprouts (results not shown). Figure 3B illustrates how in our model the ECs move toward the tumor in response to the VEGF gradient.

### ECM Proteolysis

We model the ECM using a PDE describing the evolution of a scalar field $c_{E}(\vec{x})$. MMPs degrade the ECM at a rate proportional to the ECM concentrations:

6 $$ {\partial c_E(\vec{x},t)\over\partial t}= -\delta(\sigma_{\vec{x}},0)\epsilon_{\mathrm{EM}}c_M( \vec{x},t)c_E(\vec{x},t) $$

where $c_{E}(\vec{x},t)$ and $c_{M}(\vec{x},t)$ represent the concentrations of ECM and MMPs, and *ϵ*_EM_ is a degradation constant. VEGF induces MMP-secretion by activating Notch signaling (Plaisier et al. 2004; Funahashi et al. 2011); we therefore assume that ECs secrete MMPs at a rate proportional to the local VEGF concentration,

7 $$ \begin{aligned}[b] {\partial c_M(\vec{x},t)\over{\partial t}}={}& \alpha_{\mathrm{MV}}c_V( \vec{x},t) \bigl(1-\delta(\sigma_{\vec{x}},0)\bigr)H\bigl(c_{M,\rm {max}}-c_M( \vec{x},t)\bigr) \\ &-\delta(\sigma_{\vec{x}},0)\epsilon_{M}c_M( \vec{x},t)+D_M\nabla^2 c_M(\vec{x},t), \end{aligned} $$

where $c_{V}(\vec{x},t)$ represents the concentration of VEGF, and *α*_MV_, *ϵ*_*M*_, and *D*_*M*_ are the secretion rate, decay rate, and diffusion coefficient of MMPs. The Kronecker-delta expressions state that cells only secrete MMPs at the lattice sites they cover, while the ECM is only degraded outside the cells. The heaviside step function, $H(c_{M,{\rm max}}-c_{M}(\vec{x},t))$, suppresses secretion of MMPs if the local MMP concentration exceeds the maximum concentration of $c_{M,{\rm max}}=1$. Note that in our model the nondiffusible ECM components do not decay at lattice sites that are occupied by the cells of a growing sprout. This is a simplified way to represent the balanced ECM decay and production that preserves matrix integrity near the cell.

### Numerics, Initial Conditions and Boundary Conditions of PDE

We solve Eqs. (6) and (7) numerically with a forward Euler method with fixed time step on a square lattice with the same dimensions as the one used for the CPM. We discretize the Laplacian in Eq. (7) using a five-point stencil method. To solve the interaction of the PDE and CPM, we apply a first-order operator splitting strategy: we run 15 iterations of the fixed time-step forward-Euler scheme between subsequent MCSs with Δ*t*=1. We use zero boundary conditions for Eq. (7). We initialize the simulations with a high uniform concentration ECM ($c_{E,{\rm init}}(\vec{x})=0.9$) outside the parent vessel, an intermediate concentration ($c_{E}(\vec{x})=0.5$) inside the vessel and a semicircular gradient in front of the opening in the basal membrane (Fig. 1C), which presents a biologically plausible starting situation.

### Cellular Responses to ECM Components

To model haptokinesis, we assume that the membrane protrusion rate of ECs is favored at intermediate concentrations. A reverse Gaussian describes this dependence of cell protrusion rate on ECM concentration, yielding the following haptokinesis term:

8 $$ {{\Delta H}_{\mathrm{haptokinesis}}}=-\eta\delta(\sigma_{\vec{x},0}) \biggl(-1+{ {1\over{\rho\sqrt{2\pi}}}}e^{-{{(c_E(\vec{x}')-\mu)^2}\over{2\rho ^2}}} \biggr), $$

where *η* is the haptokinesis strength, *μ*=0.5 is the intermediate ECM density with ECM densities in our model with *c*_*E*_∈[0,1]; the standard deviation *ρ* is set to a value *ρ*=0.2 to ensure diversity of cell motility over the range of available ECM values. The reverse Gaussian increases cell motility at intermediate ECM densities, while reducing cell motility at high or low ECM densities (see Fig. 2B).

Figures 3C and D illustrate the effect of haptokinesis and ECM proteolysis on the ECs. In Fig. 3C, we include the haptokinesis term (Eq. (8)) in the Hamiltonian, initiating the simulation with a uniform, high concentration of ECM components. This reduces the cellular motility and inhibits sprout formation. In Fig. 3D, the ECs also produce proteolytic enzymes (Eq. (7)), which degrade ECM components (Eq. (6)). The ECs can now break down the matrix and migrate away from the mother vessel, producing “tracks” of intermediate ECM concentration on top of which cells have highest motility.

### Cell Proliferation

Following the suggestion that cell division occurs at gaps between ECs, due to the release of contact inhibition (Ausprunk and Folkman 1977; Nelson and Chen 2003), in our model cells divide with a probability dependent on the proportion of their perimeter not bound to adjacent cells,

9 $$ P_{\mathrm{proliferation}}(i)= \begin{cases} 0 & \text{if $\rho_{i} < \rho_{\mathrm{min}}$},\\ \rho_i - \rho_{\mathrm{min}} & \text{if $\rho_{i} \geq\rho_{\mathrm{min}}$},\\ \end{cases} $$

where *ρ*_*i*_ is the ratio between cell-ECM perimeter and the total perimeter of a cell with index *σ*=*i*,

10 $$ \rho_i = {{\sum_{(\vec{x},\vec{x}')}\delta(0,\sigma_{\vec{x}'})\delta (\sigma_{\vec{x}},i)}\over{\sum_{ (\vec{x},\vec{x}' )} (1-\delta(\sigma_{\vec{x}},\sigma_{\vec{x}'}) )\delta(\sigma_{\vec {x}},i)}}, $$

with $\sum_{(\vec{x},\vec{x}')}$ summing over all pairs of adjacent sites in the lattice and *ρ*_min_, a threshold ratio. The numerator counts the number of sites of cell *i* adjacent to the ECM; $\delta(0,\sigma_{\vec{x}'})$ selects adjacent site pairs at cell-ECM boundaries, and $\delta(\sigma_{\vec{x}},i)$ selects site pairs of which $\vec{x}$ is in cell *i*. The denominator measures the perimeter of cell *i*, with $(1-\delta(\sigma_{\vec{x}},\sigma_{\vec{x}'}) )$ selecting all cell interfaces.

The proliferation step (calculation of the probability and division of cells) is carried out once every 5 MCS. For simplicity, we assume ECs divide perpendicular to their long axis (Minc et al. 2011). Because in our model cells tend to stretch parallel to the vessel, in the direction of the VEGF gradient, this assumption agrees phenomenologically with the experimental observation that endothelial cells divide perpendicular to the long axis of the structure in which they reside, which VEGF potentially regulates (Zeng et al. 2007). To divide a CPM cell, we estimate the direction of the cells’ minor axis from the cellular inertia tensor (Zajac et al. 2003; Merks et al. 2006),

11 $$ I(\sigma) = \left ( \begin{array}{cc} {\sum_{\vec{x}\in C(\sigma)} (x_2 - \overline{C}_2(\sigma))^2}& {-\sum_{\vec{x}\in C(\sigma)} (x_1 - \overline{C}_1(\sigma )) (x_2 - \overline{C}_2(\sigma))} \\ {- \sum_{\vec{x}\in C(\sigma)} (x_1 - \overline{C}_1(\sigma )) (x_2 - \overline{C}_2(\sigma))} & {\sum_{\vec{x}\in C(\sigma)} (x_1 - \overline{C}_1(\sigma ))^2}\\ \end{array} \right ), $$

with $C(i) = \{\vec{x}\in\mathbb{Z}^{2}:\sigma_{\vec{x}}=i\}$, the set of lattice sites covered by the cell with index *σ*=*i*, and $\overline{C}(i)={1\over|C(i)|}\sum_{\vec{x}\in C(i)}\vec{x}$, i.e., the center of mass of the cell with index *σ*=*i*. The division plane, $\vec{d}$, then becomes the minor axis of the cell: $\vec {d}=(I_{1,2},\lambda_{b}-I_{1,1})$, with *λ*_*b*_ the larger eigenvalue of *I* (Zajac et al. 2003). To divide, we assign a new index *σ*′ to the sites at one side of the shortest axis of the dividing cell. We then assign half the target area and target length of the parent cell to both daughter cells. We assume that the ECs grow gradually after cell division. Cell growth is implemented by increasing the target area *A*_*T*_ by 2 lattice sites and the target length *L*_*T*_ by 0.6 sites per 5 MCS. During angiogenesis, typically only stalk cells divide, but not the tip cells or the quiescent endothelial cells in the main vessel (De Smet et al. 2009). To represent quiescent ECs in the main vessel, we only allow for proliferation outside the parent vessel, at a minimum distance of one cell length from the vessel wall. We do not distinguish between tip cells and stalk cells in the present model, so all cells outside the parent vessel can proliferate.

Figure 3E illustrates the effect of including EC proliferation in our model. It allows the daughter vessel to grow toward the tumor without splitting up. This behavior of our model partly agrees with experimental observation: Sprouting can occur without proliferation, but proliferation is required for sustaining sprouting for a longer period and to grow a large enough sprout that can reach the tumor (Ausprunk and Folkman 1977; Gerhardt 2008).

### Haptotaxis

In vitro experiments have shown that gradients of ECM components can guide EC migration (Senger et al. 2002). To mimic such ECM-guided cell migration, or *haptotaxis*, an additional energy term increases the probability of pseudopod extensions toward higher ECM concentrations,

12 $$ {\Delta H}_{\mathrm{haptotaxis}}=-\varGamma\delta(\sigma_{\vec{x}},0) \biggl( {c_E(\vec{x})\over{1+s c_E(\vec{x})}}-{{c_E(\vec{x}')\over{1+s c_E(\vec {x}')}}} \biggr) $$

with $c_{E}(\vec{x})$, the local ECM density, *Γ*, the strength of the haptotactic response, and *s*, a saturation parameter. The saturation term reduces haptotaxis at high ECM concentrations. Figure 3F and the Supplementary Movie 1 illustrate the effect of haptotaxis: It increases the radial dispersion of ECs and increases the formation of vessel branches. The ECs are pulled toward freshly degraded parts of the ECM, which reduces the relative importance of the VEGF gradients.

In summary, the additional energy terms due to chemotaxis, haptotaxis and haptokines are added to the change in effective energy associated with a potential copying step according to Eq. (1):

13 $$ {\Delta H}= H_\mathrm{after}-H_\mathrm{before}+\Delta H_\mathrm {chemotaxis}+\Delta H_\mathrm{haptotaxis}+\Delta H_\mathrm{haptokinesis} $$

where *H*_after_ is the value of the Hamiltonian after the potential copying step and *H*_before_ the original value.

## Sensitivity Analysis

In order to find out which of the mechanisms of our model play a key role in sprouting angiogenesis and to study the sensitivity of the model to parameter variations, we ran simulations where we varied the parameter of interest and kept all other parameters fixed. We use the parameters for the simulation in Fig. 3F as a reference parameter set (see Table 1). For each parameter set, we simulated ten random realizations; Supplementary Fig. 1 illustrates the variation found in these simulations for the reference parameter set.

### Morphometric Measures

We define the *compactness*, or $C=A_{\mathrm{object}}/A_{\mathrm{convex\ hull}}$, of the sprout as a measure of branching (Merks et al. 2008), with *A*_object_ the area of the largest set of connected cells that is located outside the parent vessel, and $A_{\mathrm{convex\ hull}}$ the area of the convex hull of this set of cells. Thus, the compactness yields a value in the range [0,1] with 1, a convex object (usually a sprout growing straight toward the tumor), and a value approaching 0 indicating a connected, branched object. A disadvantage of this measure is that extensive branching can result in high compactness as well, with the branches filling up the space. We therefore also define the *height* of the sprout: It is the distance between the bottom of the dish and the tip of the cell closest to the tumor, disregarding cells dislodged from the main vessel. The *size* of the sprout is defined as the number of cells in the largest connected component, including those cells located in the parent vessel.

### Chemotaxis and Haptokinesis

**Fig. 4:**
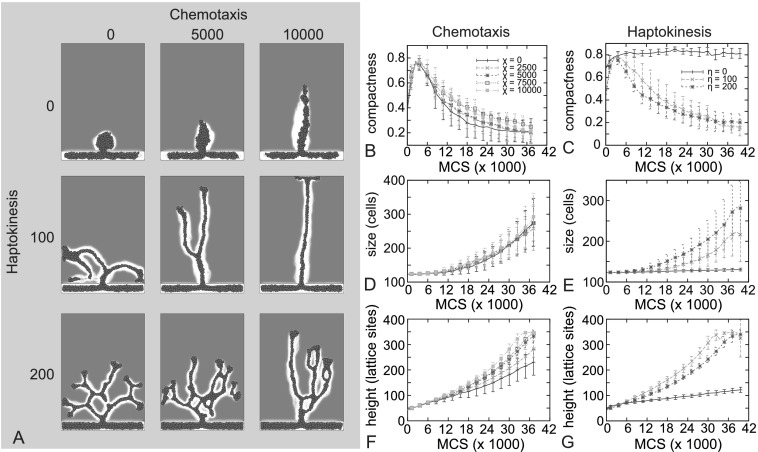
**A** Morphological response to chemotaxis parameter, *χ* (*rows*) and haptokinesis parameter, *η* (*columns*). (**B**, **D**, **F**) Compactness (**B**), size (**D**), and height (**F**) of the growing sprout with varying chemotaxis strength *χ* (see legend in panel **B**). (**C**, **E**, **G**) Compactness (**C**), size (**E**), and height (**G**) of the growing sprout with varying haptokinesis strength *η* (see legend in panel **C**). Error bars indicate standard deviation, *n*=10. Simulation results shown at 30000 MCS. All parameters except *χ* and *η* as in reference parameter set (Table 1)

We first investigated the sensitivity to the chemotaxis parameter *χ* and the haptokinesis parameter *η* (Fig. 4). The chemotactic strength does not strongly affect the compactness (Fig. 4B) or the size of the sprout (Fig. 4D). Because chemotaxis pulls the growing sprout in the direction of the tumor, the height of the sprouts increases faster with higher values of the chemotaxis parameter (Fig. 4F).

The haptokinesis parameter, *η*, affects the compactness, height and size (Figs. 4C, E, G). For zero haptokinesis, the sprouts grow slowly and do not branch, as indicated by compactnesses close to 1 (see Fig. 4A). For relatively small values of haptokinesis, the sprouts grow toward the tumor at a higher velocity, with no or few branches (Supplementary Movie 2). For higher values of haptokinesis strength, the sprout velocity decreases again because more branches are formed.

### Haptotaxis

**Fig. 5:**
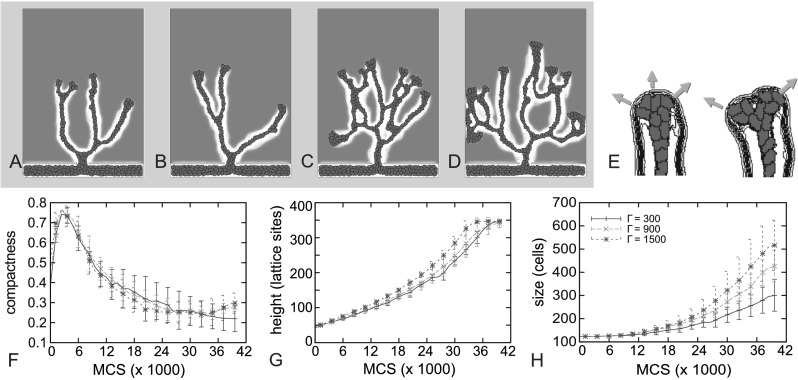
**A**–**D** Examples of growing sprouts after 30000 MCS with increasing haptotaxis strength *Γ*. **A** *Γ*=0. **B** *Γ*=300. **C** *Γ*=900. **D** *Γ*=1500. **E** Close up of sprout tip at the onset of sprout splitting. ECM gradients around the sprout tip drives cells in the tip of the sprout in opposite directions. **F**–**H** Compactness (**F**), height (**G**), and size (**H**) of the growing sprout with varying haptotaxis strength *Γ*. *Error bars* indicate standard deviation, *n*=8

The haptotaxis parameter, *Γ*, strongly affects the degree of branching of the vascular trees (see Figs. 5A–D). The compactness (Fig. 5F) and sprout height (Fig. 5G) poorly express this higher degree of branching, because the branches spread out over the available space. A slight increase of compactness is observed toward to the end of the simulation, because the continued growth (and lack of space) leads cells to fill up the space between the branches. The size of the vascular tree (i.e., the number of cells) increases with haptotaxis strength (Fig. 5H and Supplementary Movie 3), corresponding with the larger number of sprouts. Figure 5E shows what is the effect of haptotaxis on branching: As the ECM gradient around the tip drives cells in opposite directions, it causes the sprout to be pulled apart.

### ECM Degradation and ECM Density

**Fig. 6:**
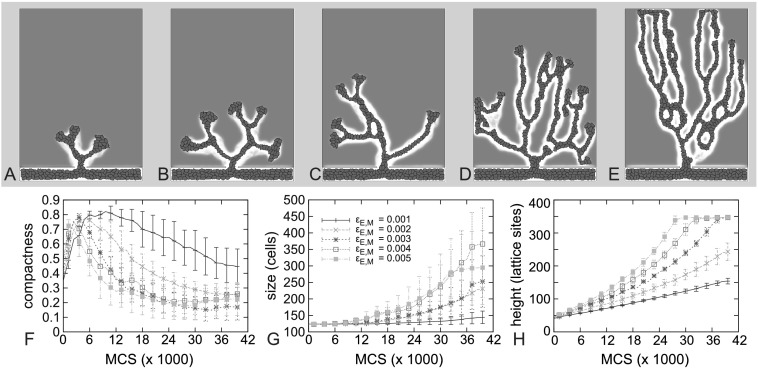
**A**–**E** Examples of growing sprouts after 30000 MCS with increasing MMP-dependent ECM degradation rate, *ϵ*_EM_. **A** *ϵ*_EM_=1×10^−3^; **B** *ϵ*_EM_=2×10^−3^; **C** *ϵ*_EM_=3×10^−3^; **D** *ϵ*_EM_=4×10^−3^; **E** *ϵ*_EM_=5×10^−3^. **F**–**H** Compactness (**F**), size (**G**), and height (**H**) of the growing sprout with varying MMP-dependent ECM degradation rate. *Error bars* indicate standard deviation, *n*=7

Next, we investigated how the rate of ECM degradation by MMPs affects angiogenic sprouting. As Fig. 6 shows, faster ECM degradation produces less compact, higher and bigger vascular sprouts, with a higher degree of branching. Slow ECM degradation produces much smaller, compact sprouts, which grow slowly toward the tumor. Variations in secretion rate of MMPs will have similar effects (results not shown).

**Fig. 7:**
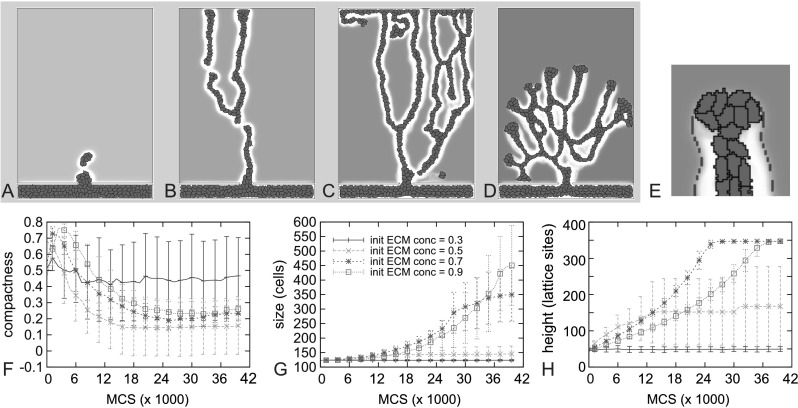
**A**–**E** Examples of growing sprouts after 30000 MCS with increasing, intial ECM concentration, $c_{E,{\rm init}}$. **A** $c_{E,{\rm init}}=0.3$; **B** $c_{E,{\rm init}}=0.5$; **C** $c_{E,{\rm init}}=0.7$; **D** $c_{E,{\rm init}}=0.9$. **E** Close up of a growing sprout in ECM field with $c_{E,{\rm init}}=0.9$. Intermediate ECM concentrations (as marked with the concentration isoline) appear at the lateral sides of the tip of the sprout, showing how haptokinesis can induce sprout splitting. **F**–**H** Compactness (**F**), size (**G**), and height (**H**) of the growing sprout with varying initial ECM concentrations. Haptotaxis strength *Γ*=1000, other parameters as in Table 1. *Error bars* indicate standard deviation, *n*=10

Figure 7 shows the effects of ECM density on the vascular tree. At low initial ECM concentrations up to to 0.3 (Fig. 7A), the sprout does not grow; ECM concentrations are below the minimum concentration for haptokinetic cell motility, and proteolysis decreases the ECM density even more. At intermediate ECM densities of around 0.5, the ECM concentration produces optimal haptokinesis, and the cells in the tip of the sprout migrate so fast that the sprout loses its coherence and branches split off (Fig. 7C). Higher ECM densities of around 0.7 (Fig. 7C) again slow down the ECs, creating a rapidly growing stable sprout. The nominal value of 0.9 used for the ECM density in our simulations (Fig. 7D) slows down cells producing shorter, more widely branched vessels. These effects are mostly due to haptokinesis (see Fig. 2B and Eq. (8)). This can be explained by the fact that intermediate ECM concentrations are mainly found at the lateral sides of the tip of the sprout, which causes cells to grow in opposite directions (Fig. 7E).

## Discussion

In this paper, we presented a cell-based model to explore the potential role of ECM-guided cell motility in angiogenesis. The model describes cell–matrix interactions on the level of individual cells. Although in this model cells can form a coherent “sprout” by chemotacting toward an external source of VEGF (Fig. 3B), only with haptokinesis, proliferation, and proteolysis do the cells in our model organize into a branched vessel tree (Fig. 3E). Branching is enhanced by including haptotaxis (Figs. 3E and 5). In haptokinesis and haptotaxis, the local concentration of ECM and the local gradients guide the velocity and movement direction of ECs. The ECs regulate the local ECM concentrations by secreting matrix-degrading MMPs, and in this way regulate their own motility and that of subsequent ECs.

We thus explored the ECs’ possible use of the ECM as a “guidance cue,” in a way similar to that proposed by Yin and coworkers (Yin et al. 2008). According to their observations in microfluidics set-ups and in absence of a preexisting ECM, Yin et al. proposed that ECs secrete collagen and change their velocity in response to local collagen concentrations. The ECs were also thought to become less sensitive to collagen concentration in response to VEGF. This would reduce the ECs’ ability to retrace paths of collagen at higher VEGF concentrations. Using an agent-based model, it was proposed that these rules allow ECs to form coherent tracks of cells navigating toward a VEGF-producing tumor, in a mechanism closely resembling one proposed previously for the formation of pheromone-mediated army ant raid patterns (Deneubourg et al. 1989).

Although the cell behaviors represented in the model of Yin et al. (2008) were based on experimental observations, they may only apply to in vitro situations in absence of external ECM materials. In vivo and in many in vitro systems (e.g., Folkman and Hauenschild 1980; Koolwijk et al. 1996) endothelial cells are embedded in an extracellular matrix, and ECs’ main effect on the ECM may be matrix degradation, not secretion. We therefore focused our study on ECM degradation. In addition, by using a multiparticle cell-based method, like the cellular Potts model, we could describe cell behavior in more detail than what is possible using continuum models (Levine et al. 2001a; Levine et al. 2001b) or agent-based formalisms that describe endothelial cells as point particles (Anderson and Chaplain 1998; Plank and Sleeman 2003; Yin et al. 2008; McDougall et al. 2006b). This allowed us to include stretch induced cell division in our model. Also, in our model the flexible shapes of the cells at the tips were required for branch splitting.

The work presented in this paper is primarily intended as an explorative study: what cellular self-organization potentially results from ECM-guided cell migration, and what role could it play in angiogenesis? Thus, the model necessarily is a strong simplification of angiogenesis in vitro or in vivo. Nevertheless, the following aspects of the model behavior agree with experimental observation. Without ECM degradation, the cells cannot invade the matrix. This model behavior agrees with studies that demonstrate that MMPs are essential for cell migration through 3D ECM matrices (Ghajar et al. 2006; van Hinsbergh and Koolwijk 2008). Furthermore, we have shown that sprout formation depends on the concentrations of matrix proteins, with vessels growing fastest at intermediate ECM densities. At low densities no sprouts will form and at very high densities the vasculature will grow at a lower rate. This phenomenon has also been observed in experimental studies (Ghajar et al. 2006; Ingber and Folkman 1989). Although it is questionable that the secretion of MMPs by all cells and the subsequent degradation around the sprout is a realistic assumption of our model, a study of extracellular proteolytic activity during angiogenesis has found that capillary sprouts are surrounded by “empty space,” resulting from fibrin degradation (Pepper 2001). In our model, such “empty space” along the stalk of the sprout is required for the formation of stable sprouts: Haptotaxis and haptokinesis lock the cells into a central zone where the ECM concentration is higher than in the immediate vicinity of sprouts, so cells cannot leave the sprout. A further realistic aspect of our model is the requirement for cell proliferation: in the first phase of angiogenesis the growth of the sprout is mainly caused by migrating cells, in a later stage proliferation is responsible for sprout growth. Without proliferation, we can reproduce branching sprouts, but they remain small in size and ECs may detach from the main sprout (Fig. 3D). Experiments show that both EC migration as well as proliferation plays a role in the formation of vessel sprouts. If proliferation is inhibited sprouts can form, but will not reach the tumor (Paweletz and Knierim 1989).

However, the model also produces unrealistic phenomenology. The growing sprouts in our simulations form bulbs at the end of branches. Cells at the tip of a sprout divide at a higher rate, because they tend to have more contact with the surrounding ECM. We could improve on this aspect of our model by reducing cell division in the very tip of the sprout, which is in agreement with the observation that mainly stalk cells located just behind the tip of the sprout proliferate. Although the model oversimplifies many aspects of angiogenesis, it illustrates some basic principles of how cell-ECM interactions can coordinate collective cell behavior during branching growth. A next step will be to differentiate between tip, stalk, and phalanx cells: Tip cells are more motile than stalk cells; they lead the sprout, navigate by extending filopodia, and invade the ECM by releasing proteases. Stalk cells follow the tip cells, and form fewer filopodia than tip cells; they proliferate and secrete ECM components. In our current model, all cells are sensitive to chemotactic and haptotactic cues; they can all proliferate and secrete MMPs and ECM components. In reality, those “tasks” are divided between tip cells and stalk cells. In addition, since proliferation is induced by VEGF (Gerhardt et al. 2003), we could improve the model not only by restricting proliferation to stalk cells, but also by increasing the probability of cell division with higher VEGF concentrations.

The ECM is now modeled as a homogenous field with initially a uniform concentration of ECM components. In reality, the matrix is highly heterogeneous with irregular concentrations of a variety of matrix components. In our model, we do not distinguish between alternative ECM proteins, like collagen, fibrin, and fibronectin. In fact, the composition of the ECM affects the ability of ECs to form networks (Dye et al. 2004) and sprouts (Kaijzel et al. 2006) in vitro. Also, the current model captures the local concentrations of ECM components, but not the fiber orientations of the ECM components as in previous studies (Bauer et al., 2007, 2009; McDougall et al., 2006a; Dallon and Sherratt, 1998). Future models will include more detailed descriptions of the ECM, and cell-ECM interactions that may change local fiber orientation and resulting cell guidance: shear stress, matrix rigidity, and the direction of matrix fibers, can all guide cells when migrating into the matrix (Li et al. 2005).

A further simplification of our model is the representation of the secretion and function of MMPs. We assumed that the secretion of MMPs does not relate to the ECM density in the vicinity of the EC. In reality, cells can fine-tune proteolysis to prevent excessive break down of the matrix. We could therefore model mechanisms that inhibit or limit proteolysis or limits proteolysis when ECM densities are low enough for invasion. Also, it was long thought that the only function of MMPs was to degrade ECM components. Recent studies, however, show that extracellular proteolysis can also regulate endothelial cell function in a more indirect way. Growth factors bound to ECM components can be released by MMPs (Hawinkels et al. 2008). Furthermore, several angiogenic growth factors require proteolytic processing to become active (van Hinsbergh and Koolwijk 2008). Proteolytic fragments of the ECM and other molecules have been reported to show regulatory activity in angiogenesis, either positive or negative. They are often called *matrikines* (van Hinsbergh and Koolwijk 2008). In our model, we could add matrix bound factors to the ECM, such as certain VEGF isoforms, which can be released or activated by MMPs. These factors will set up steep local gradients and this will certainly affect cell migration, as in related models of network formation (Köhn-Luque et al. 2011).

## Ancilliary Materials

**Supplementary Movie 1**: Example of simulation with reference parameter settings (Table 1).
**Supplementary Movie 2**: Example of simulation with high chemotaxis strength (*χ*=7500) and low haptokinesis strength (*η*=150). The other parameters are as given in Table 1.
**Supplementary Movie 3**: Example of simulation with high haptotaxis strength (*Γ*=1800), other parameters are given in Table 1.
**Supplementary Figure 1**: Ten random examples of growing sprouts after 30000 MCS with parameters as listed in Table 1.
**Supplementary Text 1**: Example of simulation with realistic parameter values and dimensions.

## Electronic Supplementary Material

Below are the links to the electronic supplementary material.

(PDF 205 kB)

(PDF 133 kB)

(MOV 1.1 MB)

(MOV 1.4 MB)

(MOV 2.6 MB)
