# Supplementary material for: A Cell-Based Model of Extracellular-Matrix-Guided Endothelial Cell Migration During Angiogenesis
Source: Bull Math Biol. 2013 Mar 15;75(8):1377–99. doi: 10.1007/s11538-013-9826-5 (PMC3738846; doi:10.1007/s11538-013-9826-5)
Supplement: Supplementary file 1 — (PDF 205 kB) [file 11538_2013_9826_MOESM1_ESM.pdf]

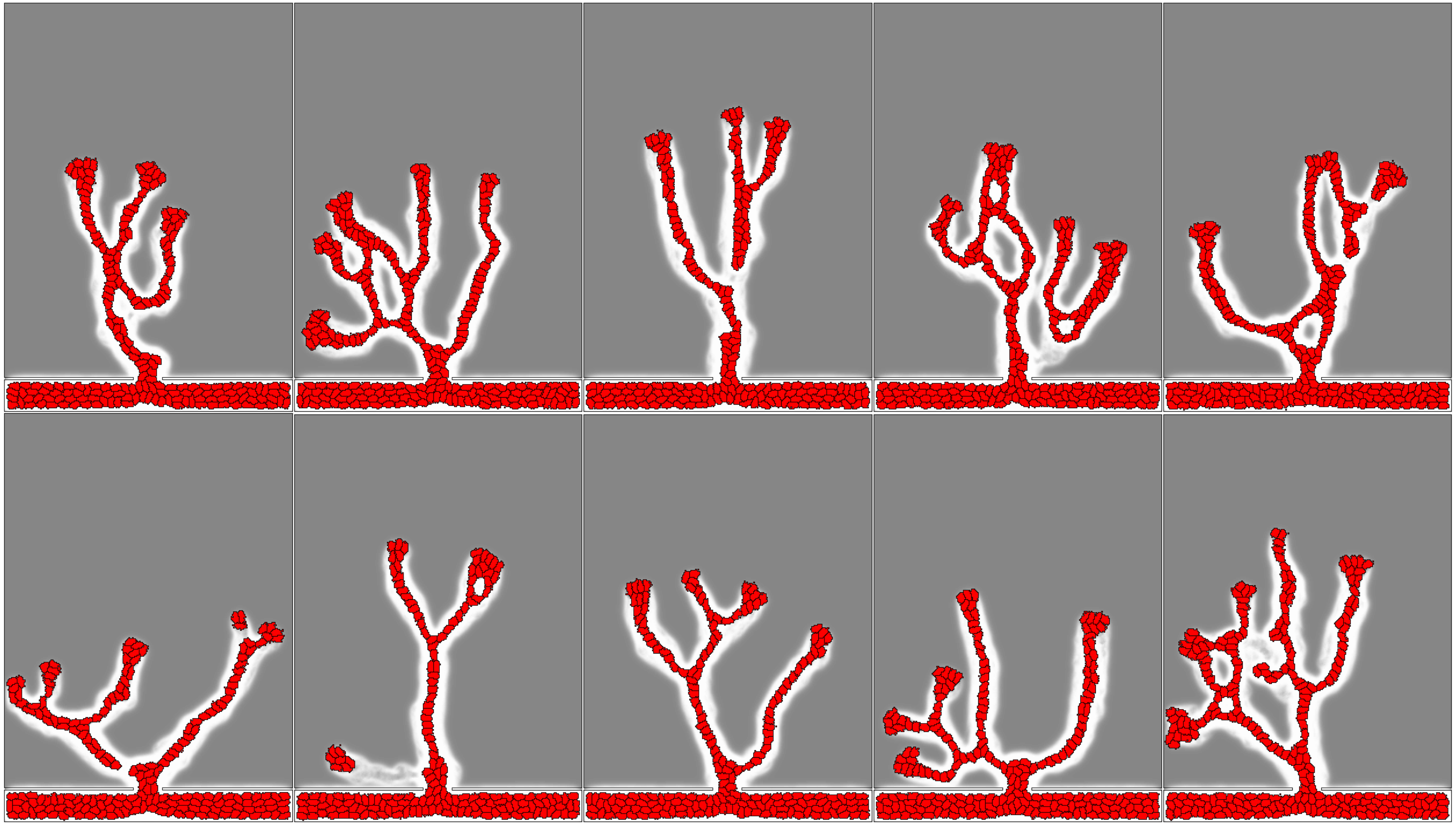

SUPPLEMENTARY FIGURE 1. Ten random examples of growing sprouts after 30000 MCS. All parameters as in reference parameter set (Table 1 in main text).
