# Supplementary material for: A Cell-Based Model of Extracellular-Matrix-Guided Endothelial Cell Migration During Angiogenesis
Source: Bull Math Biol. 2013 Mar 15;75(8):1377–99. doi: 10.1007/s11538-013-9826-5 (PMC3738846; doi:10.1007/s11538-013-9826-5)
Supplement: Supplementary file 2 — (PDF 133 kB) [file 11538_2013_9826_MOESM2_ESM.pdf]

Supplementary Text 1 to:

Daub and Merks: A Cell-Based Model Of Extracellular-Matrix-Guided Endothelial Cell Migration During Angiogenesis

Simulations with empirical parameter values

To obtain insight in the relative importance of the assumptions included in our model, in the main text we study our model for dimensionless parameter settings. In this supplementary text, we show that the main result of our simulations holds if empirical values are used where they are known. For the unknown values, we choose appropriate values for the remaining free parameters.. As we have discussed in the main text it is unclear to what extent these free parameters can correct for imprecise values of the ``known" parameter values. we present here an example of such a realistic model. Table S1 we lists the values of the parameters and their units and Figure S1 shows an example of a simulation with these empirical values.

In this set up, a lattice site represents an area of 2 μm x 2μm. The model domain is now a rectangular dish of size 500 μm x 700μm where 125 endothelial cells are placed behind a vessel wall situated at 60 μm from the bottom of the dish. The cells can migrate through a 50 μm large gap in the wall into the ECM towards the top of the dish in the direction of a tumor which we assume to be located beyond the top of the dish. The target area of the cells becomes 200 μm^2 and the target length 30 μm.

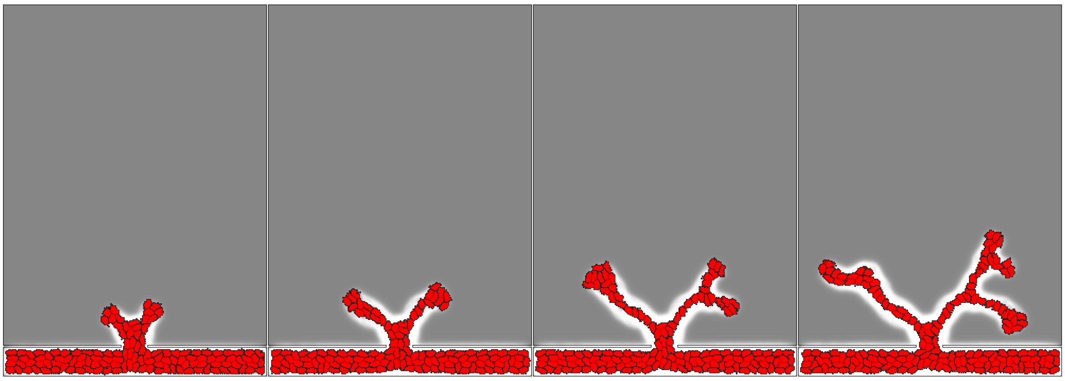

FIGURE S1. Example of a growing sprout at different time points. All parameters as stated in Table S1.

parameter	value	ref.	description
ε_{E_M}	2×10^{6} $M^{-1}s^{-1}$	[1]	Decay rate ECM by MMPs
α_{M_V}	2 s^{-1}	-	Secretion rate MMPs (we assume this value)
D_V	6×10^{-11} m^2s^{-1}	[2–5]	Diffusion coefficient VEGF
ε_V	2×10^{-4} s^{-1}	[4, 6]	Decay rate VEGF
D_M	1×10^{-12} m^2s^{-1}	[7]	Diffusion coefficient MMPs
ε_M	8×10^{-2} s^{-1}	-	Decay rate MMPs<br>We should consider the decay rate as a parameter that not only includes MMP decay, but all processes involved in restricting proteolysis to the direct neighborhood of the cell, such as MMP regulation by binding with TMPs and internalization of MMPs [8].
$c_{E,\mathrm{init}}$	4.5×10^{-4} M	[9]	Initial ECM density
$c_V(0)$	1×10^{-12} M	[10, 11]	VEGF concentration at 'top' of dish, near tumor
$c_{M,\mathrm{max}}$	1×10^{-8} M	[9]	Maximum concentration MMPs
Δt	2 sec		Time step for PDE integration<br>(with 15 steps per MCS, 1 MCS = 30 sec)
Δx	2μm		lattice spacing
χ	8×10^{13}		Chemotaxis strength
Γ	1000		Haptotaxis strength
s	7		Saturation haptotaxis
η	200		Haptokinesis strength
ρ_{min}	0.73		Threshold ratio for proliferation
λ_L	25		Area constraint
λ_A	25		Length constraint

TABLE S1. Model parameters with realistic values.

References

1. Gioia M, Monaco S, Fasciglione GF, Coletti A, Modesti A, Marini S, Coletta M (2007) Characterization of the Mechanisms by which Gelatinase A, Neutrophil Collagenase, and Membrane-Type Metalloproteinase MMP-14 Recognize Collagen I and Enzymatically Process the Two α-Chains. Journal of Molecular Biology 368:1101–1113

2. Vempati P, Popel AS, Mac Gabhann F (2011) Formation of VEGF isoform-specific spatial distributions governing angiogenesis: computational analysis. BMC Syst Biol 5:59

3. Shin Y, Han S, Jeon JS, Yamamoto K, Zervantonakis IK, Sudo R, Kamm RD, Chung S (2012) Microfluidic assay for simultaneous culture of multiple cell types on surfaces or within hydrogels. Nature Protocols 7:1247–1259

4. Chen R, Silva E, Yuen W, Mooney D (2007) Spatio–temporal VEGF and PDGF Delivery Patterns Blood Vessel Formation and Maturation. Pharmaceutical Research 24:258–264

5. Farahat WA, Wood LB, Zervantonakis IK, Schor A, Ong S, Neal D, Kamm RD, Asada HH (2012) Ensemble Analysis of Angiogenic Growth in Three-Dimensional Microfluidic Cell Cultures. PLoS ONE 7:e37333

6. Serini G, Ambrosi D, Giraudo E, Gamba A, Preziosi L, Bussolino F (2003) Modeling the early stages of vascular network assembly. The EMBO Journal 22:1771–1779

7. Collier IE, Legant W, Marmer B, Lubman O, Saffarian S, Wakatsuki T, Elson E, Goldberg GI (2011) Diffusion of MMPs on the Surface of Collagen Fibrils: The Mobile Cell Surface – Collagen Substratum Interface. PLoS ONE 6:e24029

8. Hinsbergh VWM van, Koolwijk P (2008) Endothelial sprouting and angiogenesis: matrix metalloproteinases in the lead. Cardiovasc Res 78:203–212

9. Hoshino D, Koshikawa N, Suzuki T, Quaranta V, Weaver AM, Seiki M, Ichikawa K (2012) Establishment and Validation of Computational Model for MT1-MMP Dependent ECM Degradation and Intervention Strategies. PLoS Comput Biol 8:e1002479

10. Stefanini MO, Wu FT, Mac Gabhann F, Popel AS (2008) A compartment model of VEGF distribution in blood, healthy and diseased tissues. BMC Syst Biol 2:77

11. Dabrosin C, Margetts PJ, Gauldie J (2003) Estradiol increases extracellular levels of vascular endothelial growth factor in vivo in murine mammary cancer. International Journal of Cancer 107:535–540
